# The role of OncoSnoRNAs and Ribosomal RNA 2’-O-methylation in Cancer

**DOI:** 10.1080/15476286.2021.1991167

**Published:** 2021-11-14

**Authors:** Daniela Barros-Silva, Jonathan Klavert, Guido Jenster, Carmen Jerónimo, Denis L.J. Lafontaine, Elena S. Martens-Uzunova

**Affiliations:** a Department of Urology, Erasmus MC Cancer Institute, University Medical Center, Rotterdam, The Netherlands; b Cancer Biology and Epigenetics Group, Research Center of IPO Porto (CI-IPOP) / RISE@CI-IPOP (Health Research Network), Portuguese Oncology Institute of Porto (IPO Porto) / Porto Comprehensive Cancer Center (Porto.CCC), Porto, Portugal; c Department of Pathology and Molecular Immunology, School of Medicine & Biomedical Sciences, University of Porto (Icbas-up), Porto, Portugal; d Rna Molecular Biology, Fonds De La Recherche Scientifique (F.r.s./fnrs), Université Libre De Bruxelles (Ulb), BioPark Campus, Gosselies, Belgium

**Keywords:** Ribosome, ribosomal RNA 2’-O-methylation, box C/D snoRNA, Fibrillarin, cancer biomarker, cancer diagnostics, cancer prognostics, cancer therapeutics

## Abstract

Ribosomes are essential nanomachines responsible for all protein production in cells. Ribosome biogenesis and function are energy costly processes, they are tightly regulated to match cellular needs. In cancer, major pathways that control ribosome biogenesis and function are often deregulated to ensure cell survival and to accommodate the continuous proliferation of tumour cells. Ribosomal RNAs (rRNAs) are abundantly modified with 2'-O-methylation (Nm, ribomethylation) being one of the most common modifications. In eukaryotic ribosomes, ribomethylation is performed by the methyltransferase Fibrillarin guided by box C/D small nucleolar RNAs (snoRNAs). Accumulating evidences indicate that snoRNA expression and ribosome methylation profiles are altered in cancer. Here we review our current knowledge on differential snoRNA expression and rRNA 2ʹ-O methylation in the context of human malignancies, and discuss the consequences and opportunities for cancer diagnostics, prognostics, and therapeutics.

## Introduction

Ribosomes are macromolecular nanomachines dedicated to the synthesis of proteins across all three domains of life. As such, ribosomes are essential for the maintenance and function of living cells. Their structure and composition are highly conserved, which is often seen as a testimony of their deeply rooted evolutionarily origin according to the RNA-world hypothesis. Until recently, ribosomes were often considered as invariable monolithic blocks that decode messenger RNAs (mRNAs) into proteins. However, recent biotechnological developments allowed the acquisition of genomic, transcriptomic, epitranscriptomic and proteomic data supporting the early hypothesis of ribosome heterogeneity, and suggesting the existence of compositional variation, functional plasticity, and ribosome specialization [1–5].

Ribosomes are extremely abundant, their number in mammalian cells can reach up to 10 millions [6], while ribosomal RNA (rRNA) may contribute to up to 85% of the total RNA pool [7]. Consequently, ribosomal biogenesis and function are energetically highly demanding processes that must be tightly regulated. This process requires the coordinated activity of all three RNA polymerase holoenzymes and of a large number of processing, assembly, and modification factors whose expression may be controlled by major signalling pathways (RB, p53, MYC, PI3K-AKT-mTOR) in order to fine-tune ribosomal subunit production to cellular needs and energy supplies [8]. Unsurprisingly, alterations of these ribosome assembly factors or regulatory loops are associated with different diseases, including cancer [9–11].

In cancer cells, control of ribosome biogenesis may be usurped to ensure a continuous high production of ribosomes (and subsequently, of proteins) in order to sustain unrestricted cell growth. This likely makes cancer cells more susceptible to treatments that inhibit rRNA synthesis or function, and several therapeutic approaches exploiting this property are currently developed (see below CX-5461 and BMH-21) [12–16]. Although promising, these targeted therapeutic approaches have inherent limitations as it appears that liquid cancers are better targets than solid malignancies, for reasons likely associated with tumour micro-environments and differential nutrient accessibility. In any events, a right balance of modulation of ribosome biogenesis must be found in any such therapeutic approaches, since ribosomes and protein synthesis are essential to all cells. The concept of ribosomal heterogeneity and the existence of structurally and functionally different ribosomes which may be linked to cancer-associated aberrations provide new avenues for the development of innovative cancer biomarkers and therapies for precision medicine.

In this review, we focus on the emerging role in tumorigenesis of rRNA 2ʹ-O-methylation (Nm, ribomethylation) and on the box C/D snoRNAs that guide them. We summarize evidences that specific guides snoRNAs and/or their associated modifications may vary considerably in malignant diseases. We highlight the impact of these observations for cancer diagnostics, prognostics, and therapeutics.

## 2ʹ-O-methylation of rRNA (Nm, ribomethylation)

Eukaryotic ribosomes are composed of four distinct rRNA (5S, 5.8S, 18S, and 28S) and 80 ribosomal proteins organized in two ribosomal subunits of unequal size [17,18]. The small ribosomal subunit (40S or SSU) is responsible for decoding the messenger RNA and contains the 18S rRNA and 33 ribosomal proteins. The large ribosomal subunit (60S or LSU) is involved in amino acid polymerization, it contains three rRNAs, the 5S, 5.8S and 28S and 47 ribosomal proteins [19–21].

In eukaryotic cells, ribosome biogenesis is initiated in a specialized subnuclear compartment, the nucleolus, which is now considered as a biomolecular condensate formed by liquid–liquid phase separation (LLPS) [22]. The nucleolus consists of three sub-compartments embedded in each other like ‘Russian dolls’: the fibrillar centre (FC), surrounded by the dense fibrillar component (DFC); each nucleolus containing numerous FC/DFC modules embedded in a single mass of granules, the granular component (GC). Pre-rRNA synthesis mediated by RNA polymerase I (Pol I) occurs at the interface between the FC and the DFC. The initial steps of pre-rRNA processing and ribosomal subunit assembly take place in the DFC where most box C/D snoRNA-mediated modifications are installed. The size, the shape, and the number of nucleoli per cell nucleus vary considerably in disease making it a potent biomarker, notably of the proliferative status of cancer cells [23–26]. During ribosome biogenesis, pre-rRNA transcripts are extensively modified. As a result, the mature 80S ribosomes carry 14 different types of chemical modifications at up to 228 sites [27]. Among these, 2ʹ-O-methylation (Nm, ribomethylation) at specific positions in 5.8S, 18S, and 28S rRNAs is one of the most abundant; it has been detected at up to 112 different positions in human ribosomes [28].

**Figure 1.**
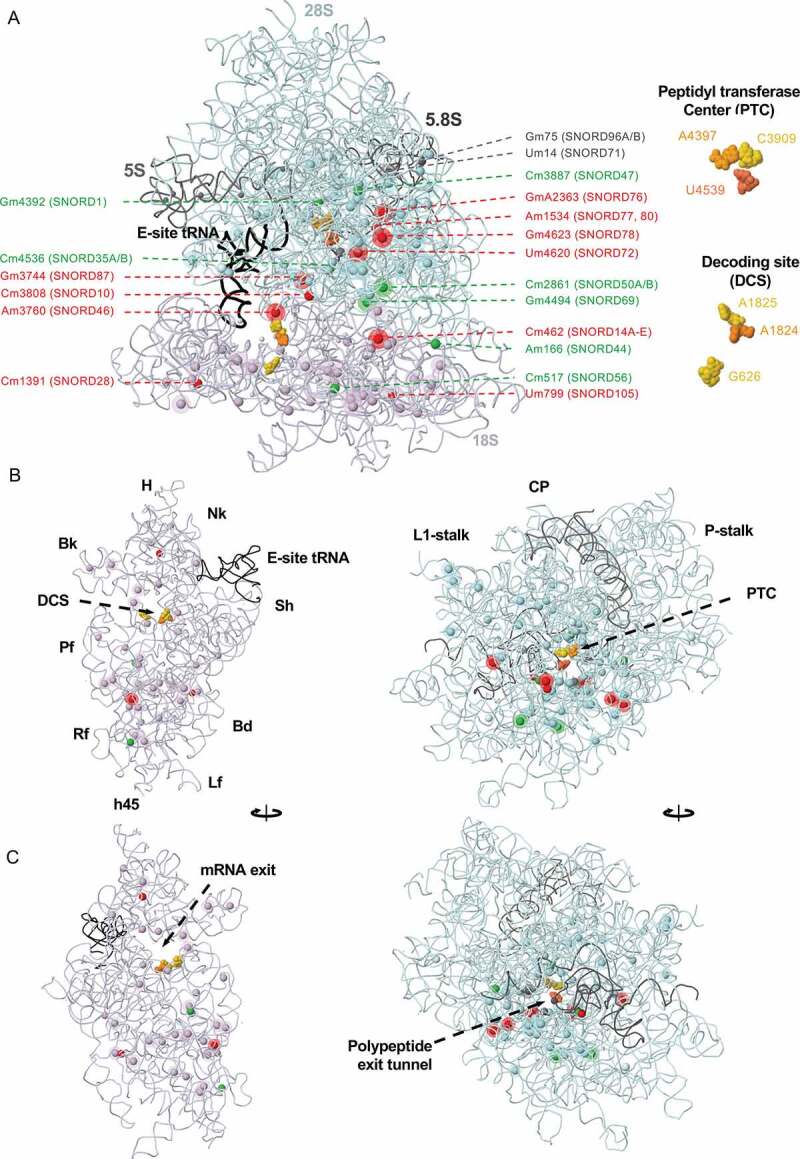
Mapping sites of rRNA 2ʹ-O methylation in space

2ʹ-O-methylation of the sugar-phosphate backbone of RNA, consists in the addition of one methyl group (-CH_3_) at the 2ʹ-hydroxyl position of the ribose moiety. Any of the four nucleosides (A, C, U, or G) may be subjected to ribomethylation at different stages during ribosome biogenesis, although most positions are understood to be modified at a rather early stage of ribosomal subunit biogenesis [29]. This is consistent with the abundant presence of Fibrillarin (FBL) in the middle layer (DFC) of the nucleolus and its role in nascent pre-rRNA sorting by LLPS from its site of synthesis (at the FC/DFC interface) into the DFC [30]. Ribomethylation increases the hydrophobicity of RNA molecules, stabilizes their structure, protects the RNA backbone from enzymatic attack, and affects potential interactions with other molecules [31]. Several ribomethylation marks are located in the proximity of functionally important regions of the ribosome, such as the decoding site (DCS) on the small subunit, and the peptidyl transferase centre (PTC) on the large one, but there are also many ribomethylation sites positioned at the periphery of the ribosomal subunits. As discussed below, these are usually more liable to variation in different conditions, including in response to p53 (ref [32].) (Fig. 1).

**Figure 2.**
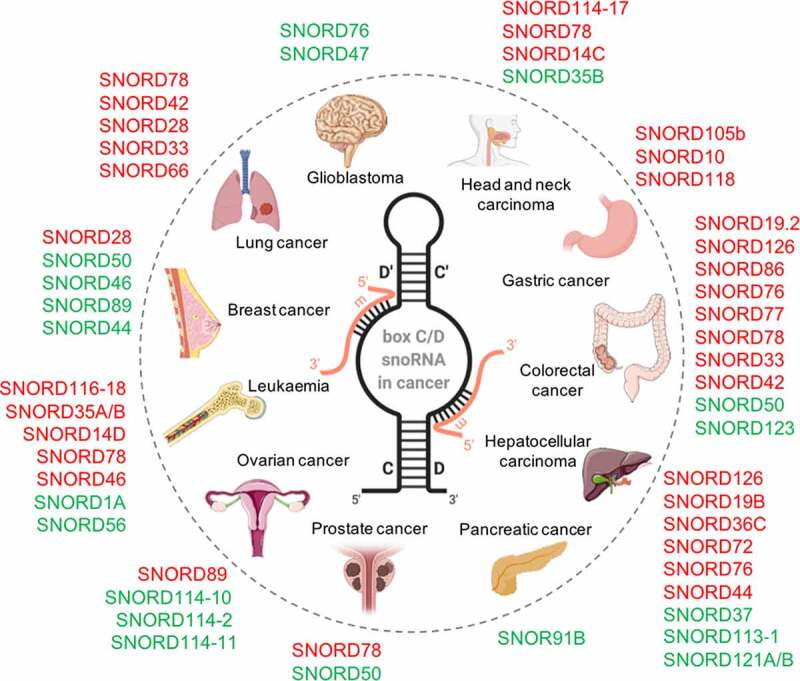
Tumorigenic role of box C/D snoRNAs

**A**, Solvent view of the human ribosome with annotated 2ʹ-O methylation sites. Some sites of 2ʹ-O methylation (solid spheres) are made constitutively; others are liable to variation and are likely regulated in health and disease (solid spheres with halos). Sites guided by snoRNAs whose levels do not vary in cancer are coloured according to their respective rRNA chain (28S, light blue; 18S, lavender; 5S and 5.8S, dark grey). Sites guided by snoRNAs upregulated in cancer are coloured red, and sites guided by snoRNAs downregulated in cancer in green (see Fig 2 for details). Beware that, as discussed in the text, there is not always a 1:1 relationship between snoRNA levels and modification levels. Residues of the peptidyl transferase centre (PTC) and the decoding site (DCS) are coloured in gold and orange and highlighted in the right panel. SNORD14A-E represents multiple snoRNAs of the SNORD14 family (*i.e*. SNORD14, 14A, 14B, 14C, 14D, 14E). **B**, Interface view of the 40S and the 60S subunits with E-site tRNA depicted. H, head; Nk, neck; Sh, shoulder, Bd, body, Lf, left foot; h45, helix 45; Rf, right foot; Pf, platform; Bk, beak; CP, central protuberance.**C**, Solvent view of the 40S and 60S subunits. This figure was generated in Jmol: Jmol: an open-source Java viewer for chemical structures in 3D. http://www.jmol.org/ and is based on the human ribosome X-ray structure at 3.60 Å resolution [21] (PDB 4UG0).

The oncosnoRNAs define a large family of non-coding small nucleolar RNAs that may act as proto-oncogenes (in red, increased levels in cancer) or tumour suppressors (in green, reduced levels). In the centre, structure of a typical box C/D snoRNA (black) base-paired to its substrate (orange). The methylated position on the substrate is located 5 nucleotides away from box D (or box D') on the snoRNA.

Interestingly, in a ribosome population, the levels of modification at the different sites susceptible to be 2ʹ-O methylated in the ribosome are not always the same [5,32–36]. Indeed, individual sites may be either fully modified (i.e. present in 100% of ribosomes in a population) or not (<100%). Fully ribomethylated sites are often positioned in the core of ribosomal subunits. Such constitutive sites show little variation among different cell types and conditions, suggesting that ribomethylation at these positions is critical for proper biogenesis and/or function of the ribosome. On the contrary, sites with partial ribomethylation often lie in the ribosome periphery and show larger variability in different physiological or pathological states; such plasticity suggests possible regulatory roles [5,32–34]. It should be noted that methylation changes observed at specific sites are often relatively modest, and, at least in some cases, could originate from interpersonal or technical variations. Systematic ribomethylation profiling efforts and functional analysis are therefore needed in order to establish the true regulatory nature of differential rRNA 2ʹ-O methylation. Nevertheless, the existence of partially ribomethylated sites is a *bona fide* argument in favour of the production of a heterogeneous population of ribosomes in cells. The possible mechanisms that influence and regulate rRNA ribomethylation, such as substrate accessibility, abundance of the antisense guides and associated proteins, *etc*., are discussed below.

## Mechanisms of rRNA 2ʹ-O methylation

In eukaryotes, the vast majority of ribomethylation marks found in cytoplasmic ribosomes are installed by a dedicated and evolutionary conserved ribonucleoprotein complex named box C/D snoRNP. An equivalent machinery prevails in Archaea where it is called sRNP [37]. A typical box C/D snoRNP is composed of one specific box C/D small nucleolar RNA (typically 65–120 nucleotides long in humans [38]) and of four associated proteins, the methyltransferase FBL, and the structural proteins NOP56, NOP58, and 15.5 kDa. The box C/D snoRNA acts as an antisense guide to target the snoRNP complex to the substrate rRNA position by forming Watson-Crick base-pairing interactions via an up to 10 nt-long sequence in the snoRNA called antisense box [39,40]. The structural integrity and nucleolar localization of the snoRNA is provided by conserved motifs termed box C (5ʹ-RUGAGA-3ʹ) and box D (5ʹ-CUGA-3ʹ) positioned close to the 5ʹ and 3ʹ ends of the snoRNA, respectively. The non-canonical base-pairing between these two motifs forms a so-called kink-turn (K-turn) structure that consists of two RNA stems separated by a short asymmetric loop with a characteristic sharp bend (kink) between the two stems. A second, internal C’/D’ pair, when it is present, forms a kink-loop (K-loop) structure made by a single stem closed by a terminal loop [41,42]. The K-turn and K-loop are important for the assembly of the snoRNP and the proper positioning of FBL with respect to its RNA substrate [41].

A few rRNA positions are 2ʹ-O-methylated by ‘stand-alone’ enzymes, which do not involve antisense guide RNAs. This is notably the case of G2922 on the yeast 25S rRNA that is modified by the 2ʹ-O-methyltransferase Sbp1 (human homolog: FTSJ3) [41,43]. In contrast, the ribomethylation of mitochondrial ribosomes follows different mechanisms resembling those found in bacteria, where individual residues are modified directly by dedicated stand-alone proteins with methyltransferase activity [28,44,45].

### Fibrillarin

Given the essential role of FBL as the only known snoRNP 2ʹ-O-methyltransferase, it can be expected that a change of FBL expression significantly affects ribosomal ribomethylation and consequently ribosome biogenesis and, possibly, performance. Indeed, FBL knockdown decreases ribosome biogenesis rates and global ribomethylation levels in human cancer cells [5]. Inversely, high levels of FBL are accompanied by changes in the rRNA ribomethylation landscape and consequent impairment of translational fidelity, possibly enabling malignant progression. Interestingly, some ribomethylation sites are more vulnerable to FBL depletion and possibly more prone to regulation than others [32]. FBL expression is under the direct control of p53 that acts as a safeguard of translational control by repressing FBL and thus preventing abnormal rRNA methylation [46]. It has been proposed that FBL-mediated modulation of rRNA ribomethylation patterns can enhance the translation of a subset of oncogenes promoting tumour initiation and progression [47]. Remarkably, most sites with documented partial ribomethylation are also vulnerable to reduced FBL levels and particularly sensitive to the absence of functional p53 [32]. Yet, specific sites are not made in absence of p53 providing an indirect evidence that ribomethylation patterns are linked to translational control and cell faith. It remains to be established however, whether single-site alterations rather than depletion of clusters of ribosomal modifications are sufficient to truly impact ribosome function.

### Box C/D snoRNAs

SnoRNAs have long been considered as house-keeping genes with ubiquitous expression. However, recent research has indicated that many box C/D snoRNAs display dynamic expression that varies throughout embryonic development, across different tissues in the body, and during tumorigenesis [48–51]. Although human ribosomes carry up to 112 methylated sites, the human genome may encode up to a thousand box C/D snoRNA genes [33,50]. Such difference in number is due in part to: **i**) the observation that a particular rRNA site may be modified by multiple alternative snoRNAs [33]; **ii**) the existence of specialized forms of box C/D-box snoRNAs involved in guiding RNA acetylation (ac^4^C) rather than 2ʹ-O methylation [52]; **iii**) the existence of a few snoRNAs involved in pre-rRNA processing rather than modification [53]; but mostly **iv**) because a large number of snoRNAs have no identified target to date. The later are referred to as ‘orphan’ snoRNAs.

Box C/D snoRNA genes (termed SNORDs) are almost exclusively intronically encoded in protein-coding and non-coding genes in higher eukaryotes [54]. Strikingly, the host genes themselves often encode proteins involved in ribosome biogenesis or function [55] implying some level of coordinated expression. When it comes to their expression patterns, snoRNAs do not represent a single homogenous group. They may be expressed specifically in particular tissues, sometimes at high levels. The host gene architecture may also vary quite substantially, *i.e*. from cases where the expression of both (host gene and snoRNA) is coupled to cases where it is completely uncoupled [51]. Expression of snoRNAs, particularly in cases of tissue-specific expression, can be uncoupled from the expression of their host gene suggesting additional levels of regulation during intron processing, snoRNA maturation, or turnover. Interestingly, most snoRNAs of this kind are encoded in long-noncoding RNAs (lncRNAs) [51]. For example, the gene encoding the evolutionary conserved lncRNA Growth Arrest Specific 5 (GAS5) hosts 10 different box C/D snoRNAs but only some of them are expressed at high levels in different cancers [56,57]. It has been demonstrated that during mouse embryonic development the expression of the GAS5-encoded SNORD78 is regulated by alternative splicing of GAS5 transcripts [58]. Nevertheless, it remains unclear at this stage if alternative splicing is the only way to control the expression of this snoRNA. Although, in the case of SNORD78, its expression levels are corelated to the methylation levels of its target site 28S-G4623, this is clearly not always the case. Therefore, it is very important to experimentally test whether there is a correlation between snoRNA and ribomethylation levels; in particular in different tissues, and in different developmental or disease states. The frequently observed cancer-related upregulation of many SNORDs does not always correspond to hypermethylation of their cognate target sites. In fact, approximately half of human SNORDs have no predictable rRNA targets, and some SNORDs have been associated with diseases that show no defects in rRNAs [59], as discussed below (section on ‘Extra ribosomal functions of snoRNAs’). So far, a causal link between box C/D snoRNAs and disease state has been demonstrated in several cases. Some of the best examples include the development of the cerebral microangiopathy leukoencephalopathy with calcifications and cysts in patients with biallelic mutations in the SNORD118 (U8) gene [60,61], the association of the congenital disease and ribosomopathy cartilage-hair hypoplasia with mutations in the RNA component of RNase MRP [62], and the embryonic development defects observed in zebrafish upon loss of snoRNAs encoded in the GAS5 locus [63].

## Cancer associated alterations of snoRNA expression

The influence of rRNA ribomethylation on ribosome biogenesis and function is yet to be fully investigated. In general, alterations in rRNA ribomethylation can impact ribosome biogenesis, protein synthesis and thereby cell proliferation [64]. Due to the initial complexity of experimental procedures associated with the molecular and functional characterization of 2ʹ-O-methylation (as discussed below, this has changed in part with the advent of deep-sequencing-based mapping approaches) a large body of cancer research investigating the role of ribosomal 2ʹ-O-methylation has instead focused on determination of snoRNA expression, for which techniques could be generally adapted from the microRNA field, and for which conventional techniques such as RTqPCR and small RNA-Seq were readily available.

Extensive high-throughput studies targeting the small transcriptome over the last 15 years (*i.e*. microarrays, tiling arrays, and more recently small RNA-Seq) revealed a surprisingly dynamic expression of snoRNA in different tissues and diseases [48,50,51]. Some box C/D snoRNAs exhibit deregulated expression in cancer, having tumour-suppressive or oncogenic functions (Fig. 2), and many are associated with major cancer hallmarks, such as sustained proliferative signalling, inhibition of cell death, and activation of invasion and metastasis in haematological malignancies and solid tumours [65,66]. Subsequently, the biomarker potential and functional role of snoRNA in tumorigenesis have been investigated in several malignancies. Below, we review cases where alterations in snoRNA expression have been documented in connection to cancer, presenting these evidences systematically by cancer types.

### Box C/D snoRNAs define novel cancer biomarkers

It is known now that alterations in box C/D snoRNA expression levels can affect several cellular processes [67], in particular tumorigenesis [50]. Several studies have reported on the performance of individual C/D-box snoRNAs or combinations of snoRNAs (and other genes) as cancer diagnostic and/or prognostic biomarkers in different malignancies (Table 1). A good example is the identification of a panel of five snoRNAs: SNORD33, SNORD66, SNORD73B, SNORD76 and SNORD78, with strong diagnostic potential in non-small cell lung carcinoma (NSCLC). The capacity of three of these snoRNAs: SNORD33, SNORD66 and SNORD76, as liquid biopsy diagnostic biomarkers for NSCLC was also established in plasma, allowing the stratification of NSCLC patients and normal subjects with 81.1% sensitivity and 95.8% specificity [68]. Remarkably, altered snoRNAs expression levels could also be robustly measured in saliva specimens from NSCLC patients and the diagnostic performance of only two snoRNAs: SNORD66 and SNORD7, outperformed that of sputum cytology with significantly higher sensitivity (74.58% *vs*. 45.76%) [69].

**Box 1.** Why should snoRNAs be considered as cancer therapeutic targets?

SnoRNAs are functionally important for ribosome synthesis and function SnoRNAs exhibit tissue- and tumour-specific patterns of expression SnoRNAs are mostly encoded intronically, implying that with proper design, their coding sequence can be manipulated at the gene level (*e.g*. using CRISPR-Cas9) without altering the expression of the host gene. SnoRNAs may also be targeted at the RNA level, using antisense oligonucleotides that may either block their Watson-Crick base-pairing capacity or lead to their degradation. SnoRNAs, and the modification they target, are emerging as biomarkers for disease (cancer) diagnosis and prognosis.

**Box 2.** Outstanding challenges

Systematically establish the involvement of snoRNAs, and the modifications they mediate in ribosome biogenesis and function Employ a pan-tissue and pan-cancer analysis to systematically assess snoRNA expression (alteration) and how it correlates to the modifications they mediate Model snoRNA mutations associated with cancer *in vitro* and *in vivo* Systematically explore non-ribosomal functions of snoRNAs Systematically examine the function of ‘orphan’ snoRNAs Explore systematically which factors control snoRNP biogenesis and function. Are there factors specific to subclasses of snoRNAs, or active in a tissue-specific fashion? Develop further and implement techniques for the systematic and quantitative mapping of rRNA modifications

**Table 1.** Box C/D snoRNA cancer biomarkers

SnoRNA name	Cancer Type	Prognostic Variable	Poor prognosis when	Hazard Ratio (Cox regression)	95% Confidence Interval	Ref.
SNORD1A	CLL	progression-free survival	lower expression	0.47	0.27–0.82	[73]
SNORD12	UM	overall survival	higher expression	2.58	1.54–4.33	[83]
SNORD35B	HNSCC	overall survival	lower expression	2.93	1.56–5.52	[78]
SNORD44	BCa	overall survival	lower expression	0.5	0.26–0.99	[57]
SNORD56	CLL	progression-free survival	lower expression	0.5	0.30–0.83	[73]
SNORD78	HNSCC	overall survival	higher expression	1.18	1.05–1.34	[78]
	NSCLC	overall survival	higher expression	1.93	1.40–3.21	[75]
SNORD87	UM	overall survival	higher expression	2.66	1.63–4.32	[83]
SNORD89	OC	overall survival	higher expression	1.40	1.08–1.82	[82]
SNORD114-17	HNSCC	overall survival	higher expression	1.30	1.11–1.52	[77]
SNORD116-18	CLL	progression-free survival	higher expression	2.49	1.46–4.27	[73]
snoRNA signature SNORD19B, SNORD36C, SNORD44	HCC	overall survival	high risk group	3.02	1.785–5.12	[80]
4 gene signature including SNORD118	GC	overall survival	high risk group	3.43	1.93–6.09	[81]
snoRNA signature including SNORD14C	SSC Larynx	relapse-free survival	high risk group	6.50	1.82–23.26	[71]

BCa, Breast cancer; CRC, Colorectal cancer; GBM, Glioblastoma; GC, Gastric cancer; HCC, Hepatocellular carcinoma; HNSCC, Head and neck squamous carcinoma; NSCLC, Non-small cell lung cancer; OC, Ovarian cancer; SCC, Squamous cell carcinoma; UM, Uveal melanoma. Hazard ratio (HR) is a measure of the effect of the tested variable (snoRNA expression) on the relative risk of reaching a study end point (e.g. cancer progression, cancer related death) in time. HR > 1 indicates increased risk, HR < 1 indicates decreased risk.

Besides their diagnostic potential, several snoRNAs have also been proposed as prognostic biomarkers. Gee *et al*. reported that breast cancer (BCa) patients presenting lower expression levels of SNORD44 had significantly lower overall survival, with 50% higher chances of lethal outcome compared to patients who presented higher expression levels of this snoRNA [57]. A transcriptome-wide profiling study revealed 13 other differentially expressed box C/D snoRNAs associated with shorter overall survival or recurrence free survival in BCa [70]. In laryngeal cancer, a four-gene classifier including the non-coding gene H19, the histone HIST1H3F and two snoRNAs: SNORD14C and SNORA16A (the later belonging to the H/ACA class involved in pseudouridine formation – the second type of abundant eukaryotic rRNA modification, not discussed here) was shown to predict shorter relapse-free survival and was particularly potent in the identification of patients at high risk of recurrence [71]. In T-cell lymphoma, SNORD71 was significantly overexpressed in cases with favourable outcome [72]. In chronic lymphocytic leukaemia high expression of SNORD116-18 and low expression of SNORD1A and SNORD56 was associated with shorter progression-free survival [73]. High expression levels of two other snoRNAs: SNORD35B and SNORD46, were reported to be significantly associated with disease relapse in paediatric B-cell precursor acute lymphoblastic leukaemia [74]. In NSCLC, upregulation of SNORD78 was associated with shorter overall survival with almost 2-fold higher risk of lethal outcome [75]. Besides SNORD78, shorter overall survival in NSCLC was also associated with the upregulation of SNORD28 and SNORD66 [76]. SNORD78 was also proposed as a prognostic biomarker in head and neck squamous cell carcinoma (HNSCC), where patients with higher SNORD78 expression levels were also at higher risk of lethal outcome [77]. The same study also found that higher expression levels of SNORD114-17 are associated with poor overall survival. Another work on HNSCC showed that SNORD35B may be used as a prognostic factor independently of established clinical risk parameters conferring almost three times higher risk of death from the disease [78]. In hepatocellular carcinoma (HCC), high expression levels of SNORD113-1 were shown to be prognostic of shorter time to relapse [79] while a snoRNA signature including, SNORD19B, SNORD36C and SNORD44 could correctly discriminate between HCC patients at high or low risk of lethal outcome [80]. Another snoRNA signature of eight snoRNAs including SNORD118 was identified in gastric cancer. The study revealed that increased snoRNA expression levels are associated with higher risk of lethal outcome [81]. Finally, shorter overall survival in ovarian cancer has been associated with elevated expression of SNORD89 [82] while a 4-snoRNA signature including SNORD12 and SNORD87 was found predictive of survival in uveal melanoma [83].

### Box C/D snoRNAs may act as tumour suppressors or proto-oncogenes

#### Tumour suppressors

One of the first examples of a tumour suppressor box C/D snoRNA was provided with SNORD50. Mutations in SNORD50, or reduction of its expression, were associated with a tumour-suppressor-like behaviour in prostate, breast and colorectal cancers [84–86]. Since then, tumour suppressor functions involving different cellular pathways have been proposed for several box C/D snoRNAs in solid tumours, (Fig. 2 and Table 2). For example, SNORD50A and SNORD50B inhibit tumour cell growth by binding directly to K-Ras in human cancer cell lines [87]. U3 or U8 (SNORD118) depletion triggers a p53-dependent antitumor surveillance response leading to p53 stabilization, cell cycle arrest, and apoptosis in breast and lung tumour cells and xenograft models [53], while SNORD47 and SNORD76 exhibit a tumour suppressor function in glioblastoma [88]. The restauration of SNORD47 expression *in vitro* and *in vivo* suppresses the invasive properties and epithelial-mesenchymal transition (EMT) characteristics of glioblastoma cells [88] while overexpression of SNORD76 in orthotopic tumour models blocks cell cycle in S-phase resulting in decreased tumour growth [89]. Interestingly, in liver cancer, SNORD76 seems to play the opposite function promoting proliferation in animal models by inducing EMT [90], while a different snoRNA, SNORD113‐1 displays tumour suppressive characteristics [79].

**Table 2.** Box C/D snoRNAs involved in oncogenic pathways

SnoRNA name	Cancer Type	Expression in cancer	Associated cellular process	Ref.
U3 or U8	BCa, NSCLC	up	Cell cycle arrest and apoptosis	^[53]^
SNORD10	GC	up	Stimulates cell growth	^[100]^
SNORD28	BCa	up	Cell proliferation	^[102]^
SNORD46	pan-cancer	up	Stimulates cell proliferation, migration, and invasion	^[50]^
SNORD47	GBM	down	Promotes invasion via epithelial-mesenchymal transition	^[88]^
SNORD50A SNORD50B	12 most common cancers	down	Cell growth by direct binding to K-Ras	^[87,128]^
SNORD72	HCC	up	Proliferation and invasion	^[98]^
SNORD76	GBM, HCC	down/up	Cell cycle block/Invasion through epithelial-mesenchymal transition	^[89,90]^
SNORD105b	GC	up	Promotes cellular proliferation, migration and invasion via ALDOA/c-Myc pathway	^[101]^
SNORD112-114 cluster	APL	up	Promotes cell growth by Rb/p16 cell cycle regulation	^[94]^
SNORD113‐1	HCC	down	Cell growth	^[140]^
SNORD114-1	AML	up	Cell cycle progression through G0/G1 to S phase transition	^[92]^
SNORD126	HCC, CRC	up	Enhances cell growth by activating the PI3K-AKT pathway via FGFR2	^[96,97]^
SNORD14D SNORD35A	AML	up	Stimulate self-renewal and cell proliferation	^[91]^
SNORD19.2 SNORD86 SNORD77	CRC	up	Metastatic spread	^[99]^
SNORD114-10 SNORD114-2 SNORD114-11	OC	down	Metastatic spread	^[95]^

AML, Acute myeloid leukaemia; APL, Acute promyelocytic leukaemia; HCC, Hepatocellular carcinoma; CRC, Colorectal cancer; GBM, Glioblastoma; GC, Gastric cancer; BCa, Breast cancer; NSCLC, Non-small cell lung cancer; OC, Ovarian cancer.

#### Proto-oncogenes

Remarkably, many studies report an oncogene function for box C/D snoRNAs revealing their oncogenic potential and their capacity to stimulate self-renewal and proliferation of cancer cells. This is the case of SNORD14D and SNORD35A in acute myeloid leukaemia (AML), where these snoRNAs are required for AML1‐ETO‐mediated leukaemogenesis [91]. Several studies in AML, also report SNORD114-1 as a candidate oncogene that promotes cell cycle progression through G0/G1 to S-phase transition. Overexpression of SNORD114-1 activates cell proliferation, while inhibition leads to the induction of cell death [92]. Overexpression of the SNORD112-114 cluster is also characteristic of acute promyelocytic leukaemia (APL), affecting Rb/p16 cell cycle regulation to promote cell growth [93,94]. Interestingly, SNORD114-2, SNORD114-10, and SNORD114-11 are downregulated in metastatic ovarian cancer compared to primary tumours [95]. In liver and colorectal cancer, SNORD126 promotes *in vitro* tumour cell proliferation and accelerated *in vivo* cell growth in a xenograft model by activating the PI3K-AKT pathway via binding to hnRNPK and regulation of FGFR2 [96,97]. SNORD72 can enhance the proliferation and invasion of liver tumour cells [98], while SNORD19.2, SNORD77 and SNORD86 contribute to metastatic dissemination in colorectal cancer [99]. In gastric cancer, knockdown of the overexpressed SNORD10 attenuates cancer cells growth [100], whereas overexpression of SNORD105b promotes cellular proliferation, migration and invasion via the ALDOA/c-Myc pathway, in cells and nude mice models [101]. In an *in vitro* breast cancer model, overexpression of SNORD28 promoted the proliferation of epithelial cells [102]. Finally, overexpression of SNORD46 was found in a wide variety of cancer cells, promoting proliferation, migration, and invasion [50]. Information summarizing these studies is presented in Table 2.

## Cancer-associated alterations of rRNA 2ʹ-O methylation

Historically, assessment of the role of rRNA ribomethylation on cellular biology and malignant transformation and progression has been largely hindered by the lack of methodology allowing the systematic quantitative profiling of the entire ribosomal 2ʹ-O-methylation landscape. The development of novel high-throughput sequencing-based RNA modification mapping techniques combined with powerful computational methods have rapidly expanded our views on the putative roles of snoRNA-mediated modifications in cancer. In recent years, the establishment of (semi-quantitative) RTqPCR protocols [103–105] and, most importantly, the development of several quantitative sequencing based high-throughput techniques (RiboMethSeq) [106–113], significantly accelerated research progress in this field. This revealed a surprisingly dynamic rRNA ribomethylation repertoire across embryonic development, cell differentiation, in specific tissues, and in cancer cells and clinical specimens (biopsies).

Cancer-associated changes in rRNA ribomethylation where reported early in breast cancer cells linking significantly increased ribomethylation at six ribosomal sites with aggressive cancer phenotype [105]. Few years later, Marcel *et al*. demonstrated that FBL is under the direct repression control of p53 and that p53 inactivation causes increase in FBL expression accompanied by significant increase of ribomethylation at seven sites in immortalized human mammary cells [46]. The same study also demonstrated that high FBL expression is an independent marker of poor cancer-specific survival and relapse-free survival, indirectly linking increased ribomethylation with cancer outcome [46]. Interestingly, the monitored rRNA ribomethylation changes were not similar among the eighteen investigated sites with some showing no variation while others demonstrating a 2- to 5-fold change in modification levels. Both studies used a semi-quantitative, reverse transcription followed by PCR technique (thereafter referred to as RTL-P; Reverse Transcription at Low deoxy-ribonucleoside triphosphate (dNTP) concentrations followed by PCR), which was based on the ability of 2ʹ-O-methylated residues to promote a reverse transcriptase ‘drop off’ at low dNTP concentration.

The development of ribomethylation sequencing (RiboMethSeq) [108], and the optimization of the protocol for use with the Illumina sequencing platform [107], demonstrated that at least one-third of the known ribomethylated sites are only partially methylated in cell line models of human malignancies (Fig. 1 and Supplementary Table 1) [5,32,33]. It was confirmed that FBL depletion in HeLa and HCT116 cells affects ribomethylation in a site-specific manner, revealing a subset of ribosomal positions with naturally variable methylation levels that are more vulnerable to changes in FBL activity [5,32]. Last year, the profiling of the first two clinical cohorts of tissue samples from breast cancer and B-cell lymphoma patients further confirmed the initial findings in cancer cell models, and demonstrated that rRNA ribomethylation profiles can discriminate between disease grades, and thus offer the potential to be utilized in the clinics for diagnostics and prognostics purposes [34,35]. Interestingly, although a significant overlap was observed, both studies identified specific subsets of ribosomal RNA residues that show different extent of ribomethylation, supporting the existence of tissue-specific and cancer-specific ribomethylation signatures. In lymphoma samples, hypomethylation was observed at positions 18S-Um354 and 18S-Cm1440 and hyper methylation at position 28S-4623(4593) [34]. In a larger BCa cohort, forty-six ribosomal sites (43.4%) exhibited high biological inter-patient variability, whereas a subset of four sites, namely 18S-Am576, 18S-Gm1447, 28S-Gm1303, and 28S-Gm4588, could discriminate between tumours with defined clinicopathological characteristics, *e.g*. tumour grade, tumour stage, hormonal and mutational (HER2, p53) status and BCa subtype. In particular, hypomethylation of 18S-Gm1447 and hypermethylation of 18S-Am576 was associated with triple negative BCa compared to luminal phenotype [35].

Interestingly, as far as we can tell, the majority of variable sites are only modified in mammalian ribosomes and are located away from the catalytic sites. In contrast, sites that are robustly modified are evolutionary conserved and located in close proximity to structural and catalytically important regions of the ribosome. It is yet unclear what is the role of individual sites with variable ribomethylation levels, but their positioning in the outer layers of the ribosome as well as the lack of conservation suggests that such sites may have evolved to reflect the increasing complexity of multicellular organisms and accommodate the specific regulatory translational needs associated with tissue and organ specialization. Another striking finding is the discovery that many of the methylation changes appear to reflect a regimen of cellular growth, characteristic of tumour cells or cells in developing embryonic tissues [34]. Collectively these results strongly suggest that the ribosomal 2ʹ-O-methylation landscape is more dynamic than previously thought and under the control of complex but yet to be established regulatory processes during normal and pathological processes, where individual sites may be subjected to specific fine-tuning involving factors beyond FBL and p53.

Although, a direct correlation between snoRNA expression and ribomethylation levels of the corresponding site is not always evident, in some cases the effect of snoRNA modulation on ribomethylation changes has been successfully investigated in more detail. For instance, in acute myeloid leukaemia, the global loss of box C/D snoRNAs with concomitant loss of ribomethylation particularly at 18S-Cm462 and 28SCm-4536(4506) is driven by MYC and the fusion oncogene AML-ETO and results in decreased self-renewal potential of leukaemic cells, while the site-specific methylation of 18S-Um116 by SNORD42A is required for leukaemia cell growth and proliferation [91,114]. In colorectal cancer, ribomethylation levels at 28S-Gm3899(3878) and 28S-Gm4623(4593) are increased via concomitant stabilization of SNORD12C and SNORD78 snoRNP complexes by the SNORD12C host gene encoding the lncRNA ZFAS1. This stabilization enhances the translation of cell cycle and metastasis-associated genes, such as EIF4A3, LMAC2, and MACC1 thereby mediating colorectal cancer proliferation [115]. Recently, it was also suggested that the MYC proto-oncogene, a major regulator of protein synthesis, specifically induced modification of a single 2ʹ-O methylated position, 18S-Cm174. Interestingly, ribosomes harbouring MYC-induced 18S-Cm174 translated more efficiently proliferation-related mRNAs with concomitant changes in cellular phenotype [116]. This is in line with earlier observations demonstrating that many snoRNAs are biologically relevant, evolutionary conserved targets under the direct transcriptional control by MYC [117].

Whether the functional consequences of altered snoRNA expression on major cancer characteristics observed in the clinics or the laboratory are directly reflecting changes in rRNA ribomethylation is yet to be demonstrated in the majority of cases. Establishing such correlation is important considering that snoRNAs, as well as fragments derived from them, may exhibit additional regulatory functions beyond their involvement in ribosome biogenesis. Some of these extra-ribosomal functions are briefly summarized below.

## Extra-ribosomal functions of box C/D snoRNAs

**Figure 3.**
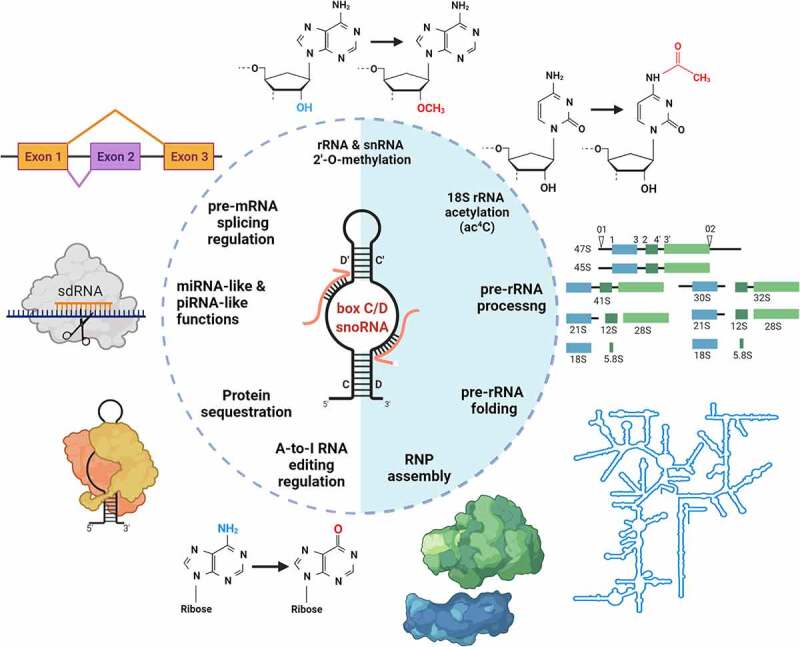
The diversity of functions of box C/D snoRNAs

In addition to their well-characterized roles in ribosome biogenesis through their involvement in pre-rRNA modification, processing, and folding, mature box C/D snoRNAs, fragments derived from them (termed snoRNA-derived RNAs or sdRNAs) [118], or even precursor snoRNA transcripts may carry additional regulatory functions (Fig. 3) (recently reviewed in [119,120]).

The schematics depicts functions of box C/D snoRNAs, or fragments derived from them (sdRNA), in RNA metabolism. The best characterized roles include RNA modification, processing, folding and ribonucleoprotein particle (RNP) assembly. Other, less conventional roles, include involvement in pre-mRNA splicing regulation, in RNA silencing, in protein sequestration, and in A-to-I editing regulation.

For example, box C/D snoRNAs have been involved in pre-mRNA splicing, editing and polyadenylation [121–125], as well as in chromatin remodelling [126,127]. Remarkably, it has also been shown that box C/D snoRNAs can influence protein function through direct binding. As mentioned earlier, the most notable example is the interaction between SNORD50A/SNORD50B and the K-Ras protein, which counteracts oncogenic signalling [87]. Consistently, SNORD50A/SNORD50B deletion and oncogenic KRAS mutation were found to co-occur significantly in multiple human malignancies [87]. More recently, it was reported that SNORD50A and SNORD50B also enhance the interaction between the ubiquitin ligase TRIM21 and its substrate GMPS subsequently influencing the interaction of GMPS with p53. Depending on p53 mutational status this interaction would either promote or inhibit malignant phenotype of breast cancer cells [128]. Another example is SNORD12B, a snoRNA predicted to target 28S-G3899(3878) [129]. SNORD12B is an oncogenic snoRNA that activates AKT-mTOR-4EBP1 signalling via nuclear partitioning of the kinase PP-1α in oesophageal squamous cell carcinoma [130].

Another example of regulation involving snoRNA/protein interaction has been demonstrated in the case of the ‘orphan’ SNORD86, which is intronically encoded in the NOP56 gene. By adopting different RNP conformation, SNORD86 acts as a sensor and ‘master switch’ controlling the levels of a limiting snoRNP core protein NOP56 [131]. Interestingly, FBL may also be regulated by a similar decoy mechanism where a nucleolar-specific lncRNA (LoNA) would recruit FBL via its snoRNA-like 3′-end to modulate its activity [132].

SdRNAs, often originating from *bona fide* snoRNAs with known ribomethylation guide function, have also been implicated in the regulation of mRNAs abundance by association with proteins from the Argonaute family conferring them miRNA-like [133,134], or piRNA-like [135] properties. For example, sdRNA-93 expression was shown to contribute to the malignant phenotype of breast cancer [134], while extensive production of sdRNAs from various snoRNAs was reported in prostate cancer [56]. Another possible role of sdRNA is as modulators of alternative splicing [38]. Interestingly, sdRNAs originating from box C/D snoRNAs are Dicer-independent [136], rarely incorporate into Ago2 complexes [137], and have a typical length of about 27 nt [56], which is closer to that of piRNAs than miRNAs. Therefore, their miRNA-like function appear limited to individual cases. Although the functional role and significance of sdRNAs is yet to be fully evaluated, it is clear that their expression in cancer tissues is altered and may carry biomarker potential. sdRNA expression signatures alone are sufficient to distinguish patients with distinct cancer types, and a subset of individual sdRNAs with tumour-immune signatures could also predict patient survival [138].

It remains to be established, whether such extra-ribosomal functions of snoRNA represent a wider but still largely unknown regulatory layer of gene expression.

## Conclusions & perspectives

Despite promising advances, a systematic effort to define the putative roles of individual snoRNAs and rRNA 2ʹ-O methylation marks in tumorigenesis and cancer progression remains to be established.

Will it be possible in the future to target ribosome biogenesis, or even may be differentially methylated ribosomes, for anti-cancer interventions? A hint that this might become possible is provided by the recent development of small-molecule inhibitors of ribosome biogenesis active on liquid malignancies. First-in-class molecules, such as CX-5461 and BMH-21 that inhibit pre-rRNA synthesis performed by RNA Polymerase I, appear to kill preferentially cancer cells and have entered multiple clinical trials worldwide [12]. Why cancer cells are more vulnerable to ribosome biogenesis modulators than non-cancerous ones is not entirely clear but it may be explained by their greater dependence on ribosomes and translation to sustain their unrestricted growth. However, clearly, cell lines are not all equally sensitive to Pol I inhibitors, and they represent only some aspects of tumour growth *in vivo* [139,140]. An encouraging example stemming from a more complex system is the use of these ribosome biogenesis inhibitors in patient-derived xenografts of late-stage prostate cancer, which are resistant to traditional treatments (*i.e*. chemotherapy and/or anti-androgens) [13,14]. Evaluation of CX-5461 in advanced phase I clinical trial is also ongoing for patients with BRCA1/2 deficient tumours (NCT02719977) [12].

RNA synthesis is only one of the many steps of ribosomal subunit biogenesis. Targeting ribosome biogenesis at other levels than RNA synthesis will undoubtedly be exploited in the future. An interesting case, is the natural alkaloid haemanthamine extracted from the bulbs of Daffodils, which targets both ribosome biogenesis in the nucleolus, activating p53 through nucleolar surveillance, and ribosome function in the cytoplasm by interfering with translation elongation [16]. Nonetheless, at this stage, there are only a few known pharmacological inhibitors of ribosome biogenesis, and most of them are highly toxic, implying they cannot be developed as drugs.

Reprogramming rRNA methylation may lead to production of specialized ribosomes displaying altered translation of specific subset of key tumour suppressor/oncoproteins and/or change in translational fidelity. Interestingly, p53-mediated alteration of rRNA methylation increased amino-acid mis-incorporation and stop codon readthrough [46]. Altered and/or heterogeneous patterns of rRNA modifications in human cancer were shown to impact tRNA and mRNA binding, affecting the intrinsic capability of ribosomes to initiate translation from internal ribosome entry site (IRES) elements that play a central role in tumorigenesis, such as in the case of c-Myc and p53 [5,105]. Thus, we can reasonably speculate that targeting specifically such oncoribosomes will offer novel and potent therapeutic opportunities to treat cancer. For many years it was possible to target with antibiotics differentially methylated ribosomes in bacteria, suggesting that theoretically this could also be possible in human. The successful use of antisense oligonucleotides targeting snoRNAs [91,115], and the rapid progress in the field of RNA-targeting therapeutics in terms of specificity and delivery may provide further opportunities for the development of a novel class of cancer drugs. Multiple arguments (listed in Box 1) support the suitability of snoRNAs as therapeutic cancer targets.

In the future, targeting oncoribosomes will likely occur as a combination therapy regimen in conjunction with small-molecule drugs that suppress other cancer-promoting pathways, or which further activates apoptosis of cancer cells. Before this can happen, several open questions remain to be addressed to understand fully how rRNA modifications are regulated, how they contribute to the biogenesis and function of ribosomes, and how important they are for normal and pathophysiological processes (Box 2). Answering these questions will inspire current and future research in ribosomal biology, and hopefully will translate into novel solutions for the clinical management of cancer and other diseases.

## Take home message

The 2ʹ-O methylation landscape of ribosomes is emerging as a new dynamic layer of biological variability. Accumulating evidence demonstrates that cancer ribosomes display specific ribomethylation signatures. The pathways and factors involved in differential modification are not understood yet, but a growing body of literature indicates that alteration of rRNA 2ʹ-O methylation is at least one of the possible ways to adapt ribosome composition, and possibly function, to the metabolic needs of tumour cells. A systematic investigation of rRNA 2ʹ-O methylation and how it correlates with snoRNA guide expressionis therefore warranted in order to fully evaluate the opportunity to develop innovative strategies targeting differentially expressed snoRNA and/or modified ribosomes in cancer and other diseases.

## Supplementary Material

Supplemental Material
